# Editorial: Neurophysiology of Silence: Neuroscientific, Psychological, Educational and Contemplative Perspectives

**DOI:** 10.3389/fpsyg.2021.675614

**Published:** 2021-04-13

**Authors:** Tal Dotan Ben-Soussan, Narayanan Srinivasan, Joseph Glicksohn, Jean-Yves Beziau, Filippo Carducci, Aviva Berkovich-Ohana

**Affiliations:** ^1^Research Institute for Neuroscience, Education and Didactics (RINED), Patrizio Paoletti Foundation for Development and Communication, Assisi, Italy; ^2^Department of Cognitive Science, Indian Institute of Technology Kanpur, Kanpur, India; ^3^Centre of Behavioural and Cognitive Sciences, University of Allahabad, Prayagraj, India; ^4^Department of Criminology, Bar-Ilan University, Ramat Gan, Israel; ^5^The Leslie and Susan Gonda (Goldschmied) Multidisciplinary Brain Research Center, Bar-Ilan University, Ramat Gan, Israel; ^6^Brazilian Research Council (CNPq), University of Brazil, Rio de Janeiro, Brazil; ^7^Neuroimaging Laboratory, Department of Physiology and Pharmacology, Sapienza University, Rome, Italy; ^8^Department of Learning, Instruction and Teacher Education, Faculty of Education, University of Haifa, Haifa, Israel; ^9^Department of Counseling and Human Development, Faculty of Education, University of Haifa, Haifa, Israel; ^10^Edmond J. Safra Brain Research Center, University of Haifa, Haifa, Israel

**Keywords:** silence, consciousness, meditation, awareness, time, neuroscience, epigenetics

The importance of silence has been emphasized in both ancient and modern traditions (Teschner, 1981; Davies and Turner, 2002; Stratton, 2015). In Eastern traditions, silence has been linked to the inner stillness of the mind, a sense of equanimity and unity (Feuerstein, 1996; Lin et al., 2008). At the same time, Western scholars such as Kierkegaard (1993) went as far as to prescribe creating silence as a remedy to the world's condition, and Wittgenstein (2002), who regarded silence as the answer to philosophy's most difficult questions, has stated that “What we cannot speak about we must pass over in silence.”

Scientifically, meditative practices have been widely investigated in the neuroscientific, psychological and contemplative fields. However, silence, which is very often a characteristic aspect of those meditative practices, has enjoyed very little focal attention in the same disciplines. The scientific study of silence-induced effects, as well as typological conceptualizations, have only sporadically appeared (Belanoff, 2001; Dénommé-Welch and Rowsell, 2017; Valle, 2019). Consequently, the purpose of the current Research Topic was to advance our understating of the subjective experience of silence, its relation to different contemplative traditions and to current theories of consciousness, its related neural mechanisms, and its possible relation to psychological outcomes, as well as educational and social perspectives.

The project was born with the realization of the first International Conference on the Neurophysiology of Silence (ICONS) held in Assisi, Italy in the summer of 2019. The Research Topic is the culmination of the effort to accumulate and present multiple approaches on silence and initiate new dialogues, and it includes both empirical and theoretical accounts, as well as reviews, for exploring silence.

Theoretical contributions include three papers which consider silence in relation to the study of consciousness without content or with minimal content: one is Srinivasan's contribution, which is an investigation on traditional statements about a state of consciousness without content in Indian tradition and its actual possibility in light of a current neurophysiological model of minimal phenomenal experience (MPE). Another paper by Paoletti and Ben-Soussan introduces the concept of silence as a tool for sensory saturation that can be hypothetically utilized to produce states of consciousness without content, discussing this hypothesis in the framework of the Sphere Model of Consciousness. In another paper, Josipovic and Miskovic addresses the differences between MPE and non-dual awareness.

Empirical contributions included four studies, utilizing diverse disciplinary and methodological approaches. Glicksohn and Ben-Soussan focus on absorption, visionary experiences and their electrophysiological correlates, pointing out that silence has a role as a creative stimulus for producing a spiritual experience. Woods et al. use evidence synthesis based on expert texts from three meditative traditions, namely Shamatha, Trascendental, and Still Meditation, with the key finding that silence has a particular connection with stillness, and the absence of concepts, mental noise, thoughts, and disturbance. Pintimalli et al. study the effects of a new meditative technique, and explore the relationship between first-person reports related to silence, space, and self-consciousness. Finally, Ben-Soussan et al. utilized neurophenomenology to explore the connection between first-person reports of silence and neuroanatomical changes.

In terms of reviews, Pfeifer and Wittmann analyse the literature and present an exploration of the perception of silence, by exposing individuals to several minutes of silence in different contexts, finding that silence increases relaxation, improves mood states, and alters the perception of time and the orientation toward the present moment. Venditti et al. review a growing body of literature exploring epigenetic changes related with different meditative practices, such as mindfulness meditation, Vipassana, Yoga, Tai Chi, and Quadrato Motor Training. Finally, Naor and Mayseless focus on the experience of solitude in the wilderness, by exploring how the wilderness solo is experienced and understood, specifically as contributing to therapeutic outcome and personal growth. They review the empirical and theoretical literature, pointing to the significance of solitude and silence, to enhance a sense of personal belonging and purpose.

In conclusion, research on silence offers many challenges, including for example, a common definition of silence (e.g., as absence of content), capturing this phenomenon in neuroscientific setups, or measuring it by scales. However, considering silence's possible benefits for the individual and societal level, overcoming such obstacles to research is highly warranted. We further propose that future studies should be conducted in the interface between psychology and education, particularly important in the current pandemic times, to explore how loneliness can be positively re-interpreted as silence and solitude and thus affect well-being differently. We wish to thank Frontiers for their support, the guest editors, authors, and reviewers for their precious time and contribution. We hope that this Research Topic will serve as another step toward integrating the literature on, as well as enrich our understanding of the beauty and utility of silence.

## Author Contributions

All authors listed have made a substantial, direct and intellectual contribution to the work, and approved it for publication.

## Conflict of Interest

The authors declare that the research was conducted in the absence of any commercial or financial relationships that could be construed as a potential conflict of interest. The handling Editor declared a shared affiliation, though no other collaboration, with one of the authors FC.
